# Experimental study on the movement of heavy metal Zn in paddy soil under different irrigation quota of reclaimed water

**DOI:** 10.1038/s41598-020-67777-x

**Published:** 2020-07-01

**Authors:** Yunfei Tuo, Cuiping Yang, Fangyuan Shen

**Affiliations:** Ecology and Environment Department, Southwest Forestry University, Kunming, 650224 China

**Keywords:** Ecology, Environmental sciences, Hydrology

## Abstract

To reveal the mechanism of heavy metal Zn migration in the irrigated paddy field reclaimed water, this study investigated irrigation quota of 75, 150, 225 and 300 m^3^/hm^2^ for three consecutive years. The results showed that with the same irrigation quota, firstly the content of Zn, its variation and the rate of change in soil increased, and hereafter decreased with the increase of soil depth, and finally become stable of reclaimed water. Study results identified that when the irrigation quota was 75, 150, 225 and 300 m^3^/hm^2^, the average content of Zn in the soil reached the maximum with the value of 9.60, 12.10, 16.75 and 18.50 mg/kg respectively at the depth of 30 cm. The average content of Zn in soil found maximum values of 13.51, 16.01, 19.02 and 20.98 mg/kg, respectively on the 120th day of cultivation. This study also identified that the content of Zn, its variation and the rate of change increased with the increase of irrigation quota at the same soil depth. Additionally, when the soil depth or plant growth time was the same, the content of Zn, its variation and the rate of change increased with the increase of irrigation quota. However, at the soil depth of 30 cm, the content of Zn in the irrigation quota of 75, 150 and 225 m^3^/hm^2^ decreased by 48.11%, 34.59% and 9.46%. The fertility time of 120 days also decreased by 35.71%, 23.81% and 9.52% respectively compared to an irrigation quota of 300 m^3^/hm^2^. All the findings are explored by a nonlinear regression under different situations and timing. The mean value of the standard error between the statistical and measured value is found insignificant. However, the correlation coefficient is found greater than 0.9400 and statistically significant. Thus, the findings by nonlinear regression reflected the migration law of soil Zn duly with soil depth and plant growth time in the rice field. This study provided theoretical support for the comprehensive treatment and ecological restoration of heavy metals to the farmland soil in China.

## Introduction

Generally reclaimed water is known as the discharge industrial and domestic sewage wastewater by the municipal sewage treatment plants after secondary treatments. Reclaimed water from irrigation is one of the important methods to solve the shortage of agricultural water consumption worldwide. India uses 2.6 m^3^ billion of reclaimed water each year for agricultural irrigation, however, Israel's reuse rate of reclaimed water is about 72% followed by Japan. In contrast, the Chinese reuse rate of reclaimed water is about 40% ~ 50%. The reclaimed water is rich with nitrogen, phosphorus, potassium and other nutrient elements for crop growths. Thus, reclaimed water in irrigation can be used to improve soil’s physical, chemical properties, aggregate structure, enhance fertility, and improve the utilization efficiency of fertilizer.

Shuhua Ru studied the migration and accumulation of heavy metals such as Cu, Zn, Pb and Cd in the soil-radish system. The results showed that the growth of radish was significantly inhibited when the concentrations of Cu, Zn, Pb, and Cd were 800, 1,000, 1,000 and 5.00 mg/kg respectively^1^. Xuebin Qi studied different irrigation mode in the reclaimed water on soil heavy metal residues for harvesting, and results showed that the total irrigation district’s soil heavy metal, Cd contents found higher than the root alternates. Accordingly, the underground drip irrigation district was found higher than the secondary sewage chlorine underground drip irrigation district. The secondary sewage ditch irrigation district was also found higher than the water irrigation district. However, the drip irrigation plot of secondary sewage treatment under total irrigation was found lower than that of under trough irrigation plot, and the ditch irrigation plot under alternate root irrigation was found lower than that of under drip irrigation^2–4^.

Zhongyang Li selected red soil, tidal soil and black soil with the content of heavy metal, Cd as level 2 study to conduct soil cultivation experiments. This study found that the content of effective Cd did not change in red soil, however, it increased significantly in tidal soil and black soil^5^. Some research works used mathematical models to simulate the adsorption and migration process of Cd in the saturated soil under drainage conditions, and results showed that different textures of soil had different retardation effects on Cd migration, and particularly, hydrodynamic dispersion had obvious effects on Cd migration in soil^6,7^. Quin and Syers studied pastures irrigated reclaimed water for 16 years in New Zealand, and the results showed that long-term use of reclaimed water in the irrigation would not cause the accumulation of heavy metals in soil with annual irrigation of 840 mm^8^. Smith et al. studied the changes of heavy metals in the soil of large green land irrigated area by the secondary treatment water between 4 to 17 years. The results showed that the accumulations of Cu, Cr, Zn, Ni, Pb and Cd in the regenerated water of irrigated soil were not exceeded the normal range, and findings were not significantly different from the contextual values of the soil^9–11^. Surdyk et al. studied irrigated tomatoes by reclaimed water for 3 years and found that the content of heavy metals in the soil was the same before and after the experiments^12^. Laidlaw used reclaimed and freshwater from a sewage treatment plant in Victoria, Australia to investigate the field irrigation of willows and used plant extractor to extract heavy metals from willow organisms. This study found that the contents of Ni, Pb, Zn, Cu, and Cd extracted from willow organisms from irrigated reclaimed water were higher than freshwater irrigation^13^.

Recently, several scholars mainly focused on reclaimed water irrigation and aimed to understand the interaction of heavy metals between irrigation and reclaimed water for Mn, Ni, Fe, Pb, Zn, Cu, Cd. For example, many research works studied the laws of migration, convection, and diffusion of various heavy metals in soil and their physicochemical models. Additionally, many research works also studied the adsorption, digestion, and accumulation of various heavy metals by soil and plants and their effects on the ecological environment. However, few studies explored the migration law of the heavy metal, Zn in the paddy field under different irrigation quotas and reclaimed water. However, few studies examined the migration law of the heavy metal, Zn in the paddy field under different irrigation quotas and reclaimed water but without considering the interaction of other soil heavy metals, unlike considering multifactor experiments. Therefore, this study did overcome earlier gaps, and experiments carried out to explore the migration law of Zn in the paddy field under different irrigation quotas and reclaimed water. This study used the parameters of irrigation quota, soil depth and rice plantation growth time as a scope. A nonlinear regression equation is utilized to provide theoretical support for comprehensive treatment and ecological restoration of heavy metals in the farmland soil.

## Material and methods

### Overview of the test area

This experiment was carried out in the reclaimed water irrigation test area at Yuanmou county (e.g. Institute of Thermal Ecological Agriculture, Yunnan Academy of Agricultural Sciences), China. The location of the test area has a hot climate with distinct dry and wet seasons. The annual average temperature is 21.90 °C with a mean temperature in the coldest month is 14.90 °C and the hottest month is 27.10 °C. The average annual precipitation is 630.78 mm. The precipitation in the rainy season accounts for more than 90% of the annual precipitation. The average annual evaporation is 3,426.30 mm which is 5.40 times of the average annual precipitation. The average annual relative humidity is 55.80%, whereas the average annual sunshine is 2,630.40 h, and the frost-free period is 350 ~ 365 days.

### Experimental treatment

Using reclaimed water from the municipal sewage treatment plant in Yuanmou county, this study carried out irrigation experiments on rice fields with different irrigation quotas for three consecutive years. The test design was mainly based on irrigation and drainage engineering design standards (GB 50288-2018). The rice irrigation water balance formula and local farmers' experience in high yield irrigation were used to set irrigation quota. Thus, the irrigation quota of reclaimed water is set as 75, 150, 225 and 300 m^3^/hm^2^. The water depth of the converted rice field area was 7.50, 15.00, 22.5 and 30.00 mm. The treatment with a 75 m^3^/hm^2^ irrigation quota was used as the test control. The experiment was designed using the orthogonal method which was set to 4 levels in total. The experimental orthogonal method replicated 3 times each level. There were 12 experimental treatments in total, and each experimental treatment cell was 20 m long and 5 m wide.

To prevent water irrigation, the cement isolation board was used in the middle of each residential area with a depth of 4.00 m and a width of 0.40 m. The experimental irrigation times were used 4 times, and the irrigation time was on April 10, May 4, May 28, and June 14 in each calendar year. The rice variety *Dian Chao 3* was transplanted on April 5 and harvested on July 10 with a growth period of 121 days. During the experiments, each plot was irrigated according to the irrigation quota set by the test plan and no freshwater mixed irrigation was conducted. Soil samples were taken from each plot every 10 days. The depth of soil drilling was 0 ~ 1.00 m, and soil samples were taken every 0.10 m. The soil samples were immediately taken back to the laboratory for the determination of Zn in the soil by atomic absorption spectrometry. The basic physical and chemical parameters of the initial soil of the paddy field under different treatments were measured before the test shown in Table 1. The heavy metal content in the reclaimed water and water quality standards for irrigation were shown in Table 2.

**Table 1:** Soil physical and chemical properties in the test area.

**Soil**	**PH**	**TOC**	**CEC**	**K**^**+**^	**Na**^**+**^	**Ca**^**2+**^	**Mg**^**2+**^	**Mechanical components (%)**		
		**mg/kg**	**cmol/kg**	**mg/kg**	**mg/kg**	**mg/kg**	**mg/kg**	** > 0.02 mm**	**0.02 ~ 0.002 mm**	** < 0.02 mm**
Red soil	7.74	15.43	10.40	3.05	75.06	70.77	47.54	54.21	22.08	23.71

**Table 2:** Heavy metal content in reclaimed water and water quality standards for farmland irrigation water.

**Water quality**	**As mg/L**	**Cd mg/L**	**Cr mg/L**	**Cu mg/L**	**Hg mg/L**	**Pb mg/L**	**Zn mg/L**
Reclaimed water	0.0028	0.0003	0.0014	0.0124	0.0113	0.006	0.005
Freshwater	0.001	0.001	0.004	0.001	0.001	0.002	0.003
Farmland irrigation water quality standard (GB5084-2005)	≤ 0.05	≤ 0.01	≤ 0.10	≤ 0.50	≤ 0.001	≤ 0.20	≤ 2.00

## Results and analysis

### Transport law of soil heavy metal Zn with soil depth in the paddy field

**Figure 1:**
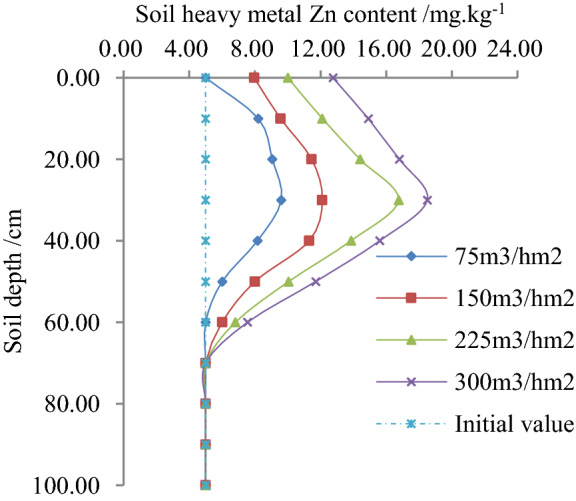
Relationship between soil heavy metal Zn and soil depth.

**Figure 2:**
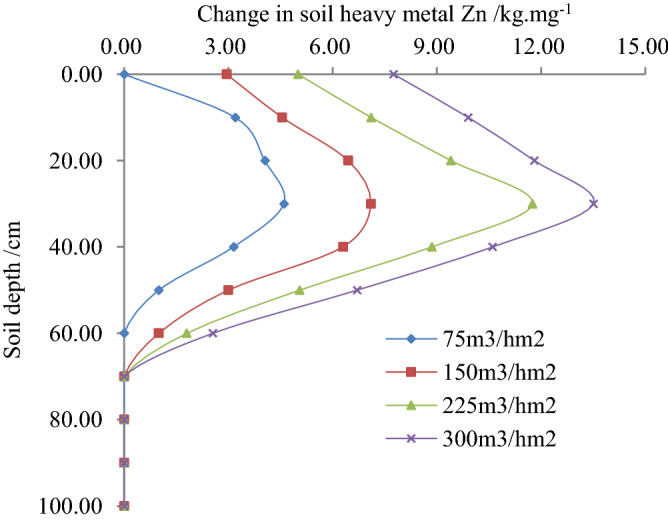
Relationship between soil heavy metal Zn variation and soil depth.

**Figure 3:**
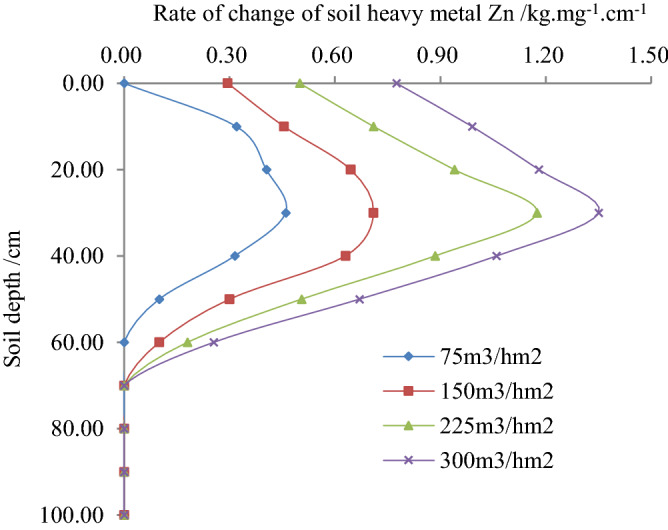
relationship between the rate of the change of soil heavy metal Zn and soil depth.

This study analyzed the data of the paddy field for irrigation tests with irrigation quota of 75, 150, 225 and 300 m^3^/hm^2^. The average value of Zn content in soil of 0, 10, 20, 30, 40, 40, 60, 70, 80, 90 and 100 cm was calculated for each soil depth measured. According to the statistical analysis of the original data, the migration law of Zn content, its variation and the rate of change with the soil depth are shown in Figs. 1, 2 and 3.

According to Fig. 1, when the irrigation quota reclaimed water was the same in different test segments, initially the content of Zn in the soil found to increase and hereafter decrease with the increase of soil depth, and finally found to be stable compared to the initial value. According to the experiments, when the irrigation quota reclaimed water was 75, 150 and 225 m^3^/hm^2^, the average content of Zn in soil decreased by 39.73%, 26.70%, and 11.76% respectively compared to 300 m^3^/hm^2^. Additionally, with the same soil depth, the content of Zn in soil found to increase with the increase of irrigation quota. Likewise, when the soil depth was 30 cm and the irrigation quota reclaimed water was 75, 150 and 225 m^3^/hm^2^, and the content of Zn was reduced by 48.11%, 34.59% and 9.46%, respectively compared to 300 m^3^/hm^2^.

It is observed from Fig. 2 that with the same irrigation quota reclaimed water, initially, the variation of Zn found to increase, and hereafter decreased with the increase of soil depth, and finally found to be stable. According to the study, when the reclaimed water irrigation quota was 75, 150, 225 and 300 m^3^/hm^2^, the soil Zn variation was the largest for the soil depth of 30 cm, and its values were 4.60, 7.10, 11.75 and 13.50 mg/kg, respectively. The changes of Zn in soil with the irrigation quota of 75, 150 and 225 m^3^/hm^2^ were found 74.53%, 50.09%, and 22.07% respectively lower than irrigation quota of 300 m^3^/hm^2^. Additionally, with the same soil depth, the variation of Zn in soil found to increase with the increase of irrigation quota. Likewise, when the soil depth was 30 cm and the irrigation quota reclaimed water was 75, 150 and 225 m^3^/hm^2^, the variation of Zn decreased by 65.93%, 47.71%, and 12.96% compared to the irrigation quota of 300 m^3^/hm^2^.

It is observed from Fig. 3, initially, the rate of the change of Zn in soil found to increase and hereafter decreased with the increase of soil depth, and finally found to be stable. According to the experiments, when the irrigation rate of reclaimed water was 75, 150, 225 and 300 m^3^/hm^2^, the change rate of Zn in the soil with a soil depth of 30 cm reached maximum, and the values were found 0.46, 0.71, 1.18 and 1.35 mg/kg.cm respectively. Additionally, when the soil depth was 30 cm, the rate of changes of Zn in the soil with the irrigation water quota of 75, 150, 225 and 300 m^3^/hm^2^ were found the highest, and the values were 0.46, 0.71, 1.18 and 1.35 mg/kg.cm, respectively. The changes of Zn in the soil were found 74.48%, 50.00%, and 21.93% lower than of 300 m^3^/hm^2^. The values are compared with the irrigation rates of the reclaimed water by 75, 150 and 225 m^3^/hm^2^. Likewise, with the same soil depth, the rate of changes of Zn in the soil found to increase with the increase of irrigation water quota. Thus, when the soil depth was 30 cm, the changes of Zn in the soil were found at 65.93%, 47.41%, and 12.96% lower than of 300 m^3^/hm^2^. The values are compared with the irrigation water quota of reclaimed water by 75, 150 and 225 m^3^/hm^2^.

The main reason was expected that the soil colloid was negatively charged, while the soil Zn^2+^ was positively charged. The two metals gathered near to the root system after adsorption, and the reclaimed water was washed by water and moved to the deep soil after irrigation. According to the analyses, the content of Zn, its variation, rate of change, soil depth and irrigation quota conformed to the nonlinear law under different irrigation quotas of reclaimed water in the paddy field^14^.

We assumed that: $x\left( {h,m} \right) = ah^{b} m^{c}$, $\Delta x^{\prime}\left( {h,m} \right) = a^{\prime}h^{{b^{\prime}}} m^{{c^{\prime}}}$, $\Delta x^{\prime\prime}\left( {h,m} \right) = a^{\prime\prime}h^{{b^{\prime\prime}}} m^{{c^{\prime\prime}}}$.

The nonlinear regression of the experimental data in Figs. 1, 2 and 3 obtained as follows:

1 $$x\left( {h,m} \right) = ah^{b} m^{c} = 4.6040h^{ - 0.3640} m^{0.3840} \;\;\;\;\;\;\;R^{2} = 0.9567$$

2 $$\Delta x^{\prime}\left( {h,m} \right) = a^{\prime}h^{{b^{\prime}}} m^{{c^{\prime}}} = 0.3380h^{ - 0.6210} m^{0.8970} \;\;\;\;R^{2} = 0.9507$$

3 $$\Delta x^{\prime\prime}\left( {h,m} \right) = a^{\prime\prime}h^{{b^{\prime\prime}}} m^{{c^{\prime\prime}}} = 0.0349h^{ - 0.6209} m^{0.8921} \;\;\;\;\;R^{2} = 0.9469$$

In types: $x\left( {h,m} \right)$—Content of heavy metal Zn in soil, $mg/kg$; $\Delta x^{\prime}\left( {h,m} \right)$—Variation of heavy metal Zn in soil, $mg/kg$;$\Delta x^{\prime}\left( {h,m} \right) = \left| {x\left( {h,m} \right) - x\left( {h_{0} ,m_{0} } \right)} \right|$;$\Delta x^{\prime\prime}\left( {h,m} \right)$— The rate of the change of soil heavy metal Zn, $mg/(kg \cdot cm)$;$\Delta x^{\prime\prime}\left( {h,m} \right) = {{\Delta x^{\prime}\left( {h,m} \right)} \mathord{\left/ {\vphantom {{\Delta x^{\prime}\left( {h,m} \right)} h}} \right. \kern-\nulldelimiterspace} h}$;$x\left( {h_{0} ,m_{0} } \right)$—Initial value of soil heavy metal Zn, $mg/kg$;$a,b,c,a^{\prime},b^{\prime},c^{\prime},a^{\prime\prime},b^{\prime\prime},c^{\prime\prime}$—fit coefficient; $h$—Soil depth, cm; $m$—irrigating water quota, m^3^/hm^2^.

Correlation analysis was carried out for Eqs. (1) - (3) respectively. The standard errors of $a,b,c$ were found 0.6680, 0.0580 and 0.1030, and the values of $a,b,c$ estimated with 95% confidence intervals were (− 0.7850, 9.9920), (− 0.4810, − 0.2470) and (0.1770, 0.5910). The standard errors of $a^{\prime},b^{\prime},c^{\prime}$ were 0.5460, 0.1260 and 0.2860. The values of $a^{\prime},b^{\prime},c^{\prime}$ estimated with 95% confidence intervals were (− 0.7640, 1.4410), (− 0.8760, − 0.3660) and (0.3190, 1.4750). The standard errors of $a^{\prime\prime},b^{\prime\prime},c^{\prime\prime}$ were 0.0560, 0.1259 and 0.2861, respectively, and the values of $a^{\prime\prime},b^{\prime\prime},c^{\prime\prime}$ estimated with 95% confidence intervals were (− 0.0780, 0.1480), (− 0.8750, − 0.3660) and (0.3150, 1.4690).

The correlation coefficient of Eqs. (1) - (3) was greater than the critical correlation coefficient of 0.9400, and their standard errors were insignificant, however, the correlation coefficient passed the significance test and statistically significant. By taking logarithms of both sides of the regression Eqs. (1) - (3), this study found that initially the content of Zn in soil changes and conversion rates increased, and hereafter decreased with the increase of soil depth, and finally found to be stable. The changes in Zn contents are compared with the increase of the irrigation quota. The findings were consistent with the rule of change shown in Figs. 1, 2 and 3. According to the statistical analysis, the mean relative error between the standard and measured value of Zn contents, its variation and rate of changes were found 8.909%, 5.221% and 6.096%, respectively. The findings showed that Zn contents in soil, its variation and rate of changes reflected the migration law accordingly with soil depth and irrigation quota in the paddy field.

### The migration law of soil heavy metal Zn with rice plant growth time

**Figure 4:**
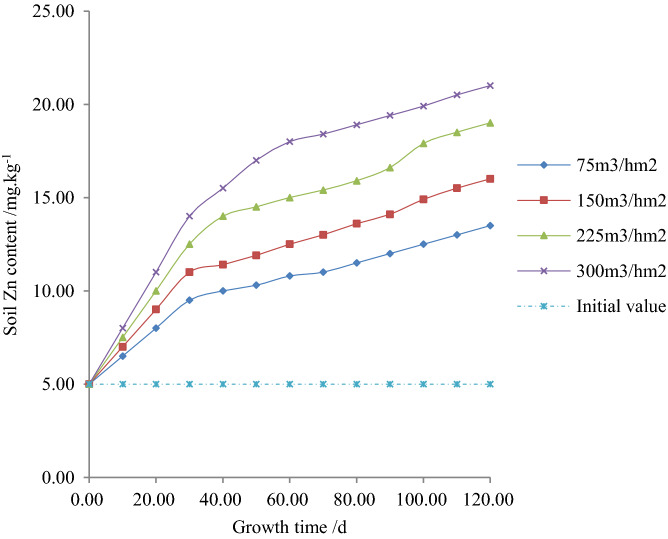
Relationship between soil heavy metal, Zn content and plant growth time.

**Figure 5:**
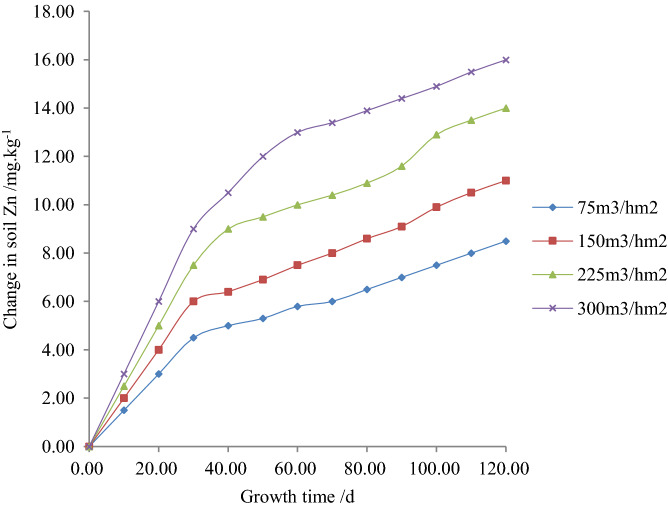
Relationship between the change of soil heavy metal, Zn and plant growth time.

**Figure 6:**
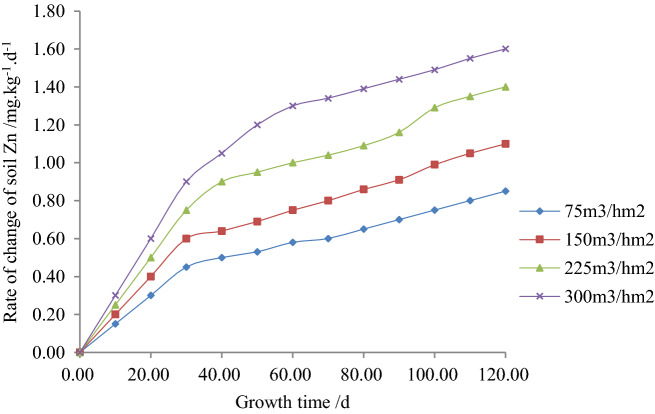
Relationship between the rate of change of soil heavy metal, Zn and plant growth time.

Figures 4, 5 and 6 respectively show the relationship between the content of Zn, its quantity change and the rate of changes with plant growth time under different irrigation quotas of reclaimed water in the paddy field.

According to experiments and Fig. 4, it is shown that when the irrigation quota of reclaimed water was the same, the content of Zn in soil increased with plant growth time. It is obvious that when the fertility time was 120 days, the content of Zn in soil reached the maximum with the irrigation water quota of 75, 150, 225 and 300 m^3^/hm^2^. The values of Zn in such experiments were found 13.51, 16.01, 19.02 and 20.98 mg/kg. During the whole growth period, the average content of Zn in soil was decreased by 35.32%, 25.01% and 11.99% when the irrigation quotas of reclaimed water were 75, 150 and 225 m^3^/hm^2^ compared to 300 m^3^/hm^2^. Furthermore, when the plant growth time was the same, the content of Zn in soil increased with the increase in irrigation quota. According to the experiments, when the fertility period was 120 days, the content of Zn in soil was decreased by 35.71%, 23.81%, and 9.52% when the irrigation quotas of reclaimed water were 75, 150 and 225 m^3^/hm^2^ respectively compared to 300m^3^/hm^2^.

According to experiments and Fig. 5, it is revealed that when the irrigation quota of reclaimed water was the same, the quantity change of Zn in soil increased with plant growth time. It is obvious that when the fertility time was 120 days, the content of Zn in soil reached the maximum with the irrigation water quota of reclaimed water of 75, 150, 225 and 300 m^3^/hm^2^, and the values were found 8.49, 10.98, 14.01, and 16.03 mg/kg respectively. However, the changes of Zn with the irrigation quota of reclaimed water of 75, 150 and 225 m^3^/hm^2^ were found 51.54%, 36.51%, and 17.49% lower compared to 300 m^3^/hm^2^. Nevertheless, with the same plant growth time, the quantity change of Zn increased with the increase in irrigation quota. Finally, it is obvious that when the fertility time was 120 days, the changes of Zn with the irrigation quota of reclaimed water of 75, 150 and 225 m^3^/hm^2^ were found 46.88%, 31.25%, and 12.50% lower than those in 300 m^3^/hm^2^.

According to experiments and Fig. 6, it is obvious that when the irrigation quota of reclaimed water was the same, the rate of the change of Zn in soil increased with the plant growth time. It is obvious that when the fertility time was 120 days, the content of Zn in soil reached the maximum with the irrigation water quota of reclaimed water of 75, 150, 225 and 300 m^3^/hm^2^ and the values were found 0.84, 1.09, 1.38 and 1.58 mg/(kg∙d) respectively. However, when the irrigation quotas of reclaimed water were 75, 150 and 225 m^3^/hm^2^, the rate of the change of Zn in soil was found 51.59%, 36.56%, and 17.57% lower than that of 300 m^3^/hm^2^. Nevertheless, with the same plant growth time, the rate of change of Zn in soil increased with the increase in irrigation water quota. Finally, it is obvious that when the plant growth time was 120 days and with irrigation water quota of 75, 150 and 225 m^3^/hm^2^, the rate of the change of Zn in soil decreased by 46.88%, 31.25%, and 12.49% respectively compared to 300 m^3^/hm^2^.

This study confirmed that the amount of Zn absorbed by the rice root system under irrigation reclaimed water was less. However, due to the increase of irrigation time, Zn in the soil found to increase with the plant growth time. According to the analyses, the content of Zn and its plant growth time, rate of the quantity change and irrigation quota confirmed nonlinear law under different irrigation quota of reclaimed water.

We assumed that: $x\left( {t,m} \right) = dt^{e} m^{f}$,$\Delta x^{\prime}\left( {t,m} \right) = d^{\prime}t^{{e^{\prime}}} m^{{f^{\prime}}}$ and $\Delta x^{\prime\prime}\left( {t,m} \right) = d^{\prime\prime}t^{{e^{\prime\prime}}} m^{{f^{\prime\prime}}}$.

The nonlinear regression of the experimental data in Figs. 4, 5 and 6 obtained as follows:

4 $$x\left( {t,m} \right) = dt^{e} m^{f} = 0.6310t^{0.3230} m^{0.3430} \;\;\;\;R^{2} = 0.9875$$

5 $$\Delta x^{\prime}\left( {t,m} \right) = d^{\prime}t^{{e^{\prime}}} m^{{f^{\prime}}} = 0.0618t^{0.5149} m^{0.5480} \;\;\;\;\;R^{2} = 0.9780$$

6 $$\Delta x^{\prime\prime}\left( {t,m} \right) = d^{\prime\prime}t^{{e^{\prime\prime}}} m^{{f^{\prime\prime}}} = 0.00601t^{0.5151} m^{0.5479} \;\;\;\;R^{2} = 0.9786$$

In types: $x\left( {t,m} \right)$—Content of heavy metal Zn in soil, $mg/kg$; $\Delta x^{\prime}\left( {t,m} \right)$—Variation of heavy metal Zn in soil, $mg/kg$;$\Delta x^{\prime}\left( {t,m} \right) = \left| {x\left( {t,m} \right) - x\left( {t_{0} ,m_{0} } \right)} \right|$;$\Delta x^{\prime\prime}\left( {t,m} \right)$—The rate of the change of soil heavy metal Zn, $mg/(kg \cdot d)$;$\Delta x^{\prime\prime}\left( {t,m} \right) = {{\Delta x^{\prime}\left( {t,m} \right)} \mathord{\left/ {\vphantom {{\Delta x^{\prime}\left( {t,m} \right)} t}} \right. \kern-\nulldelimiterspace} t}$;$x\left( {t_{0} ,m_{0} } \right)$—Initial value of soil heavy metal Zn, $mg/kg$;$d,e,f,d^{\prime},e^{\prime},f^{\prime},d^{\prime\prime},e^{\prime\prime},f^{\prime\prime}$—fit coefficient; $t$—Plant growth time, d; $m$—irrigating water quota, m^3^/hm^2^.

The correlation analysis was conducted for Eqs. (4) - (6) respectively. The standard errors of $d,e,f$ were found 0.1380, 0.0280 and 0.0350 and the values estimated with 95% confidence intervals were (0.3530, 0.9080), (0.2660, 0.3800) and (0.2740, 0.4130). The standard errors of $d^{\prime},e^{\prime},f^{\prime}$ were found 0.0100, 0.0210 and 0.0240, and the values estimated with 95% confidence intervals were (0.0420, 0.0820), (0.4730, 0.5580) and (0.4990, 0.5970). The standard errors of $d^{\prime\prime},e^{\prime\prime},f^{\prime\prime}$ were found 0.0010, 0.0218 and 0.0237, and the values estimated with 95% confidence intervals were (0.0040, 0.0080), (0.4730, 0.5580) and (0.4990, 0.5970).

The correlation coefficient of Eqs. (4) - (6) was greater than the critical correlation coefficient 0.9700, and their standard errors were insignificant, however, the correlation coefficient found statistically significant. By taking logarithms of both sides of a regression Eqs. (4) - (6), this study found that initially, the contents of Zn in soil and conversion rates increased with the increase of plant growth time and irrigation quota. The findings were consistent with the rule of change shown in Figs. 4, 5 and 6. According to the statistical analysis, the mean value of the relative error between the standard statistical and measured value of Zn content in the soil, its variation and the rate of change was 3.0390%, 0.2774%, and 2.9574%, respectively. The findings showed that Zn content in the soil, its variation and rate of change reflected the migration law duly with plant growth time and irrigation quota in the paddy field.

## Discussion

This study considered paddy field irrigation tests with irrigation quota and examined soil heavy metal zinc with soil depth and rule of change. However, other heavy metals such as Mn, Ni, Fe, Pb are excluded from the study experiments due to the study scope and limitation of the explorations. The findings are tested by multivariate statistical analysis and nonlinear regressions. We conducted irrigation experiments three consecutive years on rice fields with reclaimed water with irrigation quota of 75, 150, 225 and 300 m^3^/hm^2^ respectively. The findings from this experiment are consistent with the conclusions of Shengwei Wang and Giuseppe Protano^15,16^. We found that when the irrigation quota of reclaimed water was the same, initially the content of Zn in the soil, its variations and the rate of change increased and hereafter decreased with the increase of soil depth and finally became stable. Additionally, the content of Zn in soil, its variation and the rate of change increased with the plant growth time. With the same soil depth, this study confirmed that the content of heavy metal Zn, its variation and the rate of change increased with the increase of irrigation quota.

With the same plant growth time, the content of Zn, its variations and the rate of change in soil increased with the increase of irrigation quota. The findings are consistent with the research conclusions of Bingyi Xie^17,18^. This study also found that when the soil depth was 30 cm, the average content of Zn in soil with irrigation quota of 75, 150, 225 and 300 m^3^/hm^2^ reached the maximum and the average content of Zn reached the maximum on 120th day of the growth period. The findings are consistent with the research conclusions of Duanping Xu and Nasrin Jalilvand^19,20^. This study confirmed that the transport of Zn in the paddy soil was not only influenced by the irrigation system such as water quota, irrigation time but also influenced by the accumulation and leaching of heavy metals in the soil and the adsorption of rice roots. However, the reclaimed water still found to contain a certain amount of harmful heavy metals. The adsorption effect of rice by different kinds of other harmful heavy metals found varies greatly. However, as long as the quality standard of reclaimed water was strictly controlled, the heavy metals brought into the soil by irrigation did not affect the balance of heavy metals in the soil environment. It is noticeable currently that reclaimed water replaces freshwater as the source of farmland irrigation. Its application prospect is broad nowadays and has great significance for agricultural irrigation.

## Conclusion

This study has drawn the following conclusions on the migration law of heavy metal Zn in the paddy soil by field experiments:

1. With the same irrigation quota and reclaimed water, the content, variation, and the rate of Zn in soil initially increased and hereafter decreased with the increase of soil depth, and finally became stable. The content of Zn in soil, its variation and the rate of change increased with the plant growth time. At the soil depth of 30 cm, the average content of Zn in soil reached the maximum at the irrigation rate of 75, 150, 225 and 300 m^3^/hm^2^, with the values of 9.60, 12.10, 16.75 and 18.50 mg/kg respectively. On the 120th day of the plant growth time, the average content of Zn in soil reached the maximum value, which was 13.51, 16.01, 19.02 and 20.98 mg/kg, respectively.
2. With the same soil depth or plant growth time, the content, variation, and the rate of Zn in soil increased with the increase of irrigation quota. At the soil depth of 30 cm, the content of Zn with irrigation quota of 75, 150 and 225 m^3^/hm^2^ decreased by 48.11%, 34.59%, and 9.46% respectively compared to 300 m^3^/hm^2^, and also decreased by 35.71%, 23.81% and 9.52% respectively by 120 days from the plantation.
3. Under different irrigation quota, content, variation, and the rate of Zn in the paddy field are tested statistically. The average relative error between the standard statistical and measured value by the nonlinear regression between the soil depth and plant growth time is also estimated which is found insignificant. However, the correlation coefficient is found greater than 0.9400 and statistically significant.

The results showed that Zn followed the migration law of heavy metal in the paddy field with soil depth and plant growth time under different irrigation quota. The outcomes are explored in this study by field experiments that will support a comprehensive treatment and ecological restoration matters of heavy metals to the farmland soil in China and elsewhere with similar ecology settings.
